# *Lawsonia inermis* essential oil: extraction optimization by RSM, antioxidant activity, lipid peroxydation and antiproliferative effects

**DOI:** 10.1186/s12944-019-1141-1

**Published:** 2019-11-14

**Authors:** Asma Elaguel, Imen Kallel, Bochra Gargouri, Ichrak Ben Amor, Bilel Hadrich, Ezeddine Ben Messaoud, Radhouane Gdoura, Saloua Lassoued, Ahmed Gargouri

**Affiliations:** 1 Laboratoire de recherche Toxicologie- Microbiologie Environnementale et Santé (LR17ES06), Faculté des Sciences de Sfax, Po Box 1171, 3000 Sfax, Tunisie; 2 Unité de Biotechnologie et Pathologies, Institut Supérieur de Biotechnologie de Sfax, University of Sfax, Sfax, Tunisia; 3 Unité de Biotechnologie des Algues, Biological Engineering Department, National School of Engineers of Sfax, University of Sfax, Sfax, Tunisia

**Keywords:** *Lawsonia inermis*, Essential oil, Antioxidant activity, Cytotoxicity, Lipid peroxidation

## Abstract

**Background:**

The present study was focused on the optimization of yield of the essential oil extraction from leaves of *Lawsonia inermis*, and the determination of chemical composition, antioxidant activities, and lipid peroxydation and antiproliferative effects.

**Methods:**

Henna essential oil (HeEO) were extracted by hydrodistillation; the identification of the chemical composition were done by GC/MS method. HeEO was analyzed for antioxidant power in: (1) chemical system by the DPPH test, the ABTS test and the total antioxidant activity test; and (2) in biological system by lipid peroxydation tests (MDA and DC) in cells culture. The cytotoxicity effects of HeEO were assessed using MTT assay against Raji and HeLa cell lines.

**Results:**

The optimal extraction yield was 6.8 g/100 g d.b. HeEO showed a remarkable anti-oxidant activities including DDPH (42%), ABTS (87%) and the power of ammonium phosphomolybdate (2992 ± 230 mg of HeEO by equivalent to 1 mg of vitamin C in terms of total antioxidant power).

**Conclusion:**

Beyond notable antioxidant activities of the HeEo, our results showed a significant decrease in the production of ERO in the Raji cell line. The anti-tumor power of the Henna essential oil shows an interesting cytotoxicity effect (IC_50_ at 0.26 μg/mL for Raji and at 1.43 μg/mL for HeLa) with a total mortality percentage reaching 60%, for both.

## Background

Medicinal plants are part and parcel of human society to combat diseases, from the dawn of civilization. *Lawsonia inermis* (henna) is a member of the family of *Lythraceae* which consists of about 500 species, extensively increase in tropical regions with moderately few species in temperate regions [1]. This plant is generally considered as a native of Africa and Asia. Major producing countries are Sudan, Egypt and India [1–3].

Additionally, henna leaves have been widely used for centuries in the Middle East, the Far East and Northern Africa in cosmetics: in dyeing hair, nails, hands and in textile. These properties are due to the strong binding of a lawsone to hair and skin and to the naphthoquinone compound derived from henna [1, 3–6].

Literature shows also that henna is provided with many interesting proprieties: in fact, not tropic [7], analgesic, antipyretic, anti-inflammatory [8, 9], antibacterial [10, 11] and anti-immunomodulatory activities [12] were reported. As well, *Lawsonia inermis* has other potent pharmacological effects as an anti-tumor and anti-tuberculosis [13], anti-parasitic [14]**,** and anti-trypanosomal [15].

In Tunisia, the culture of *Lawsonia inermis* L. is artisanal and semi-industrial depending to region in south of country. It was the main speculation of coastal oases [16]. Many years ago, this crop covered about 55% of the total area, then; is cultivated in 2007 on only 13% of the areas. Haddad [17], showed that Gabes henna is known for its quality throughout the Arab world. Farmers harvest it three times a year, two months apart, between June and November. Actuality yang generation widely used them again in cosmetic or tattoo and also to medication essor.

The aim of this study was to optimize extraction of essential oil from *Lawsonia inermis* leaves, and to search an antioxidant activity, lipid peroxydation and cytotoxic effects.

## Methods

### Samples

In this study aerial parts of plants *Lawsonia inermis* (*henna)* were collected in mature period: stage green color of leaves and presence of small flowers in June 2018 from south Tunisia. Leaves were recuperated and dried at room temperature for 4 to 6 days until constant mass.

### Optimization of hydrodistillation conditions

Air-dried *L. inermis* leaves were hydrodistilled using a Clevenger-type apparatus to recuperate the essential oils for 3 h with a solid-liquid ratio of 150 g/600 mL. The distilled essential oils were dried over sodium chloride. Different concentrations were used in this step. Many washings were done with hexane solvent. The recuperated oils were stored at + 4 °C.

Table 1 presents the used levels of drying, washings and salt concentration. The adopted experimental conditions were investigated using the Box–Behnken design (Table 2). The extraction yield (g/100 g d.b.) was determined using the following eq. (1):

1 $$ Y=\frac{mass\ of\ essential\ oil}{mass\ of\ used\  dry\  matter}\times 100 $$

**Table 1:** Used levels of the experiments

Factor	Coded symbol	-1	0	+ 1
Drying (%)	x_1_	0	50	100
Washings	x_2_	3	4	5
Salt concentration (g/L)	x_3_	175	262.5	350

**Table 2:** Experimental conditions defined by Box–Behnken design

Runs	Drying (%)	Washings	Salt concentration (g/L)	Yield (g/100 g d.b.)
1	0	3	262.5	4.837
2	100	3	262.5	0.244
3	0	5	262.5	6.822
4	100	5	262.5	1.873
5	0	4	175	5.522
6	100	4	175	0.997
7	0	4	350	5.775
8	100	4	350	1.618
9	50	3	175	1.183
10	50	5	175	2.879
11	50	3	350	1.136
12	50	5	350	2.692
13	50	4	262.5	1.992
14	50	4	262.5	1.650
15	50	4	262.5	1.820

The extracted essential oil yield (Y) can be modeled as function of different factors (x_i_). The adopted eq. (2) was tested statistically:

2 $$ \hat{Y}={a}_0+\sum {a}_i\cdot {x}_i+\sum {a}_{ij}\cdot {x}_i\cdot {x}_j+\sum {a}_{ii}\cdot {x}_i^2 $$

Where $\hat{Y}$: fitted value of the dependent variable (response); *a*_0_: constant; *a*_*i*_, *a*_*ii*_, *a*_*ij*_: coefficients of factors and interactions; *x*_*i*_: coded value of factors.

The design of experiments and the different statistical analysis were carried out with Statistica 12 Software, Copyright® Stat Soft, Inc. 1984–2014.

### GC/MS composition

The *L. inermis* essential oil was analyzed using an Agilent-Technologies 6890 N Network GC system using the protocol described by Zarai et al. [18]. A sample of 1.0 μL was injected, using split mode (split ratio, 1:100). The composition was reported as a relative percentage of the total peak area. The identification and authentication of the henna essential oil (HeEO) compounds was determined using a comparison of their retention times to n-alkanes, and their mass spectra compared to published data and spectra of authentic compounds (Wiley and NIST Library).

### Antioxidant capacity assays

#### Phosphomolybdenum assay

Essential oil samples (100 μL) were mixed with 1 mL of the phosphomolybdenum reagent (600 mM sulfuric acid, 28 mM sodium phosphate, 4 mM ammoniummolybdate [19]. Then, the mixture was incubated at 95 °C during 90 min and cooled to room temperature. Subsequently the absorbance was measured at 695 nm. In order to estimate the percentage of molybdenum reduced by tested essential oil, a standard curve was constructed using ascorbic acid. EC_50_ (mg/mL) corresponds to the effective concentration at which the total antioxidant activity (TAA) at 50% and was obtained by interpolation from linear regression analysis. As a positive control, the ascorbic acid was used. The values are presented as the means of triplicate assay.

#### 2,2-Diphenyl− 1-picrylhydrazyl (DPPH) free radical scavenging activity assay

The antioxidant activity of essential oil was estimated by monitoring its ability in quenching the stable free radical DPPH. The radical scavenging activity of essential oil against DPPH free radicals was measured using the method of Clarke et al. [20] slightly modified as follows: 20 μL of appropriately diluted samples or Vitamin C solutions was added to 190 μL of DPPH solution (100 μM). The mixture was shaken vigorously and allowed to reach a steady state at room temperature for 30 min. Staining of DPPH was determined by the absorbance measuring at 517 nm with a Beckman spectrophotometer. All determination was approved out in triplicate. Ascorbic acid was used as a positive control. The DPPH radical scavenging activity was calculated according to the following eq. (3):

3 $$ I\ \left(\%\right)=\frac{A_0\left(\mathrm{total}\ \mathrm{DPPH}\right)-{A}_1\left(\mathrm{sample}\right)}{A_0\left(\mathrm{total}\ \mathrm{DPPH}\right)}\times 100 $$

where A_0_ was the absorbance of the total DPPH (blank, without extract) and A_1_ the absorbance of the sample.

#### ABTS radical scavenging activity assay

The antiradical activity was performed by the ABTS^+^ free radical decolorization assay as developed by Re et al. [21]. The 2,2-azino-bis-3-ethylbenzothiazoline-6-sulfonic acid (ABTS) was prepared as aqueous stock solution (7 mM). The ABTS radical cations (ABTS^+^) were produced by the reaction of the ABTS stock solution with 2.5 mM of ammonium persulfate methanolic solution. The reaction mixture is incubated in the dark for 16 h at room temperature. Then, the solution is diluted to an absorbance of 0.7 ± 0.02 at 734 nm to form the work reagent. The reaction mixtures containing 100 μL of the sample at different concentrations and 900 μL of reagent were incubated at 30 °C for 6 min. The antioxidant power of each sample was expressed as the inhibition percentage (I %) calculated according to the following formula (4):

4 $$ I\ \left(\%\right)=\frac{{\mathrm{A}}_0\left(\mathrm{total}\ \mathrm{ABTS}\right)-{\mathrm{A}}_1\left(\mathrm{sample}\right)}{{\mathrm{A}}_0\left(\mathrm{total}\ \mathrm{ABTS}\right)} \times 100 $$

where A_0_ was the absorbance of the total ABTS (blank, without extract) and A_1_ the absorbance of the sample.

### Determination of the antiproliferative activity

#### Human cancer cell line (HeLa et Raji)

The two tumor tested cell lines used in this study were HeLa and Raji cell lines. HeLa is a transformed line expressing the HPV18 virus (human Papiloma virus). This adherent line is obtained from tumor cells from cancer of the cervix of a 31-year-old woman [22] and Raji is a human Burkitt’s lymphoma-derived cell line, harboring the latent form of EBV cycle [23].

#### Cell line culture

The two cell lines (HeLa and Raji) were grown in RPMI 1640 medium (Gibco) supplemented with 10% (vol/vol) fetal calf serum (FCS) and 2 mM L-glutamine in tissue culture flasks (Nunc). They were passaged twice a week and kept at 37 °C in a humidified atmosphere of 95% air and 5% CO_2_.

#### MTT test

The proliferation rates of HeLa and Raji cells after treatment with essential oils were determined by the colorimetric 3-(4,5-dimethylthiazol-2-yl)-2,5-diphenyl tetrazolium bromide (MTT) assay. The yellow compound MTT is reduced by mitochondrial dehydrogenases to the water-insoluble blue compound formazan, depending on the viability of cells. Two cell lines (4 × 10^4^ in each well) were incubated in 96-well plates for 24 h in the presence or absence of essential oil. 20 μL of MTT solution (Sigma) (5 mg mL^− 1^ in PBS) were added to each well. The plate was incubated for 4 h at 37 °C in a CO_2_-incubator. 100 and 80 μL of medium was removed.

### Determination of capacity of lipid peroxydation

#### Induction of oxidative stress

TPA (12-O-Tetradecanoyl-phorbol-13-acetate) treatment: for the induction of the lytic cycle, 3 × 10^6^ cells were stimulated with 8 nM TPA for 2 h, when the cells were in logarithmic phase growth, usually 48 h after placing them in culture. The cells were washed two times with Phosphate buffer saline (PBS) and further incubated for 48 h in fresh culture medium [23].

Preparation of cell extracts: cells were centrifuged at 3000 rpm for 10 min. The pellet was resuspended in 500 μL of deionized water and lysed by five cycles of sonication during 20s at 37% (Sonisc, vibracell).

#### Malondialdehyde (MDA) determination

For evaluation of MDA production rate, thiobarbituric acid-reactive species (TBARs) assay was used. Adherent cells were detached using trypsin/EDTA solution and centrifuged at 3000 rpm for 10 min. The pellet was resuspended in 500 μL of deionized water and lysed by ten cycles of sonication during 20 s at 37% (Sonisc, vibracell). 350 μL of cell lysate are added to 700 μL reagent (thiobarbituric acid) TBA / (trichloroacetic acid) TCA. The result of this reaction is the appearance of a compound: MDA-(TBA)_2_ of pink color, whose intensity is measured at 532 nm. The mixture is put at 95 °C for 15 min. After centrifugation at 3000 rpm for 10 min, the MDA is assayed in the supernatant by measuring the optical density with the spectrophotometer (Biochrom, Libra S32) at 532 nm. This is calculated from a calibration curve determined from a standard solution of 1,1,3,3-tetraethoxypropane (1,1,3,3 PET) [24].

#### Conjugated dienes (CD)

Conjugated diene level was evaluated as described by Kurien and Scofield [25] with modification. 25 μL of cells lysat were extracted with 3 mL chloroform/methanol (2:1, v/v). After centrifugation at 3000 rpm for 15 min, 2 mL of organic phase was transferred into another tube and dried at 45 °C. The dried lipids were dissolved in 2 mL of methanol and absorbance at 233 nm was determined. It corresponds to the maximum absorbance of the extracted compounds.

## Results

### Optimization of essential oil extraction

Table 2 presents the essential oil extraction yield’s (Y). The range of the yields of extracted essential oil from the henna was 0.244–6.822 g/100 g d.b. Many experimental conditions give us extraction yields > 1.6 g/100 g d.b.

Table 3 presents the Student Test, comporting the comparison between the coefficient of each factor and interaction and the corresponding standard error.

**Table 3:** Student test of the different terms suggested for the essential oil extraction yield model

Factor	Coefficient	Standard Error	t	*p*
Constant	2.965	0.073	40.569	< 0.0001
x_1_	−2.278	0.090	− 25.450	< 0.0001
x_2_	0.858	0.090	9.590	< 0.001
x_3_	0.080	0.090	0.894	0.412
x_1_^2^	−0.782	0.066	−11.875	< 0.0001
x_2_^2^	−0.029	0.066	−0.446	0.674
x_3_^2^	−0.047	0.066	−0.707	0.511
x_1_ × x_2_	−0.089	0.127	−0.703	0.513
x_1_ × x_3_	0.092	0.127	0.727	0.500
x_2_ × x_3_	−0.035	0.127	−0.277	0.793

### The composition of henna essential oil by GS / MS

The results of the GC / MS are shown in Table 4, which reveals the presence of at least thirty of components in *L. inermis* leaves. Monpoterpene hydrocarbons were the main class of constituents with 81.40% included the α-limonene (55.06%), β–limonene (24.06%) and β–myrcene (2.28%), followed by the linalool (2.41%). The produced essential oil was characterized by a higher percentage of monoterpene hydrocarbons.

**Table 4:** Composition of the essential oil of Henna by GS / MS

Family	Compound	Composition (%)	Retention time (min)	Level of identification (%)
Monoterpene hydrocarbon	α- limonene	55.06	9.150	99
	β-limonene	24.06	8.420	99
	L-limonene	0.4	9.799	97
	β–ocimene	0.91	9.595	96
	β–myrcene	2.28	7.952	97
	β –thujone	0.26	11.441	96
	α –pinene	0.16	6.400	97
	β-pinene	0.54	7.500	97
	bicyclogermacrene	0.15	21.486	98
Phtalate	Di-phthalate	3.05	40.904	91
Alcohol	Linalool	1.58	11.027	97
	α–linalool	0.83	10.665	91
	Linalool oxide	0.88	10.228	91
	Dodecanol	0.18	19.406	90
	Epimanool	0.18	33.068	78
Terpene	Linalylacetate	1.25	15.307	91
	Geranylacetate	0.87	18.720	91
	Linalyl propionate	0.81	13.589	91
	Camphor	0.27	11.916	98
	Linalyl	0.26	18.215	91
Sesquiterpeniccarbides	Germacrene	0.95	21.124	99
Sesquiterpene	β -caryophyllene	0.81	19.572	99
	α –humulene	0.32	20.423	98
Ketone	Bi-cycloheptanone	0.61	12.187	98
	2-naphtalenone	0.16	28.576	96
Aldehyde	Caprinicaldehyde	0.60	13.973	87
AlcoholicSesquiterpene	Veridiflorol	0.44	23.784	99
Sesquiterpenealcoholic	Farnesol	0.39	23.151	94
Monoterpenecarbides	Camphene	0.13	17.967	60
Alcane	isotetradecane	0.14	19.052	70

### Antioxidant potential

#### Total antioxidant activity (TAA)

The ammonium phosphomolybdate potency in the present study was of the order of 2992.21 ± 230.17 mg of HeEO equivalent to 1 mg of vitamin C (ascorbic acid) in terms of antioxidant capacity.

#### The scavenging activity for DPPH radicals

DPPH molecules that contain a stable free radical have widely been used to evaluate the radical scavenging ability of antioxidants. The free radical scavenging activities of HeEO, was assayed by using DPPH (Fig. 3). At all the tested concentrations, *Lawsonia inermis* essential oil showed a considerable anti-radicular effect attending 42%.

#### The scavenging activity for ABTS

The total antioxidant activity of a molecule is deduced by its ability to inhibit the ABTS^+^ blue-green cationic radical by transforming it into colorless ABTS^+^ in the presence of proton derived from an antioxidant [21].

The anti-radical activity of HeEO with respect to the ABTS^+^ radicals was evaluated spectrophotometrically by following the reduction of this radical in comparison with a positive control of 6-hydroxy-2,5,7,8-tetramethylchroman- 2-carboxylic acid (TROLOX). The antioxidant activity test results of the ABTS^+^ radical by the essential oil of the leaves of *Lawsonia inermis* at 0.5 mg/mL attend 80% (Fig. 4).

### Cytotoxicity assay

A potential effect of different concentrations (0.78 to 12 μg/mL) of henna essential oil against HeLa (Fig. 5a) and (0.078 to 1.25 μg/mL) of Raji cell lines were studied (Fig. 5b).

### Effect of *Lawsonia inermis* essential oil on lipid peroxydation activity

#### Malondialdehyde (MDA) marker

The exploration of the biological antioxidant activity of HeEO was carried in the Raji human cell line (Fig. 6). Cells were cultured with or without addition of HeEO for 48 h.

#### Conjugated dienes (CDs) marker

Figure 7 shows the influence of the TPA on the Raji line.

## Discussion

The obtained essential oil extraction’s yield values can be considered as very important results compared to the latest disposable literature: 0.23% / 450 g [26] and 0.82 v/w [27] of oil. Considering the Student test (Table 3), it is clear that the drying, in linear term (x_1_) and in quadratic term (x_1_^2^), had according to the most two important coefficients: which is confirmed by a very highly significant influence (*p* <  0.0001) according to the tested dependent response (extracted essential oil yield, Y). Moreover, the second most highly significant influence is relative to the number of washings (*p* <  0.001). However, the salt concentration factor (x_3_) has not influence on the yield and there were no interactions inside studied factors (*p* > 0.05).

**Fig. 1:**
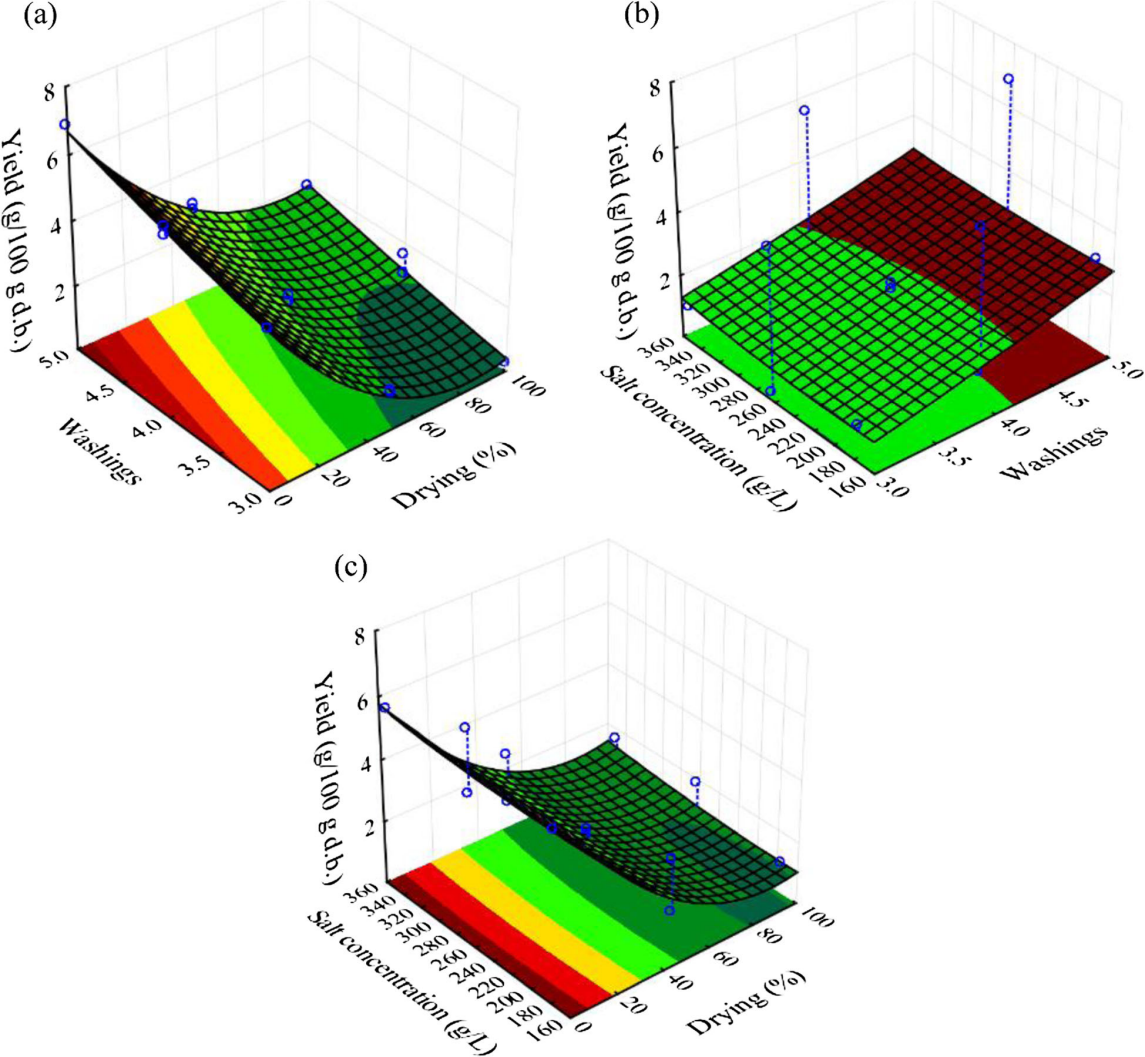
Extracted essential oil yield as function of drying and washings (**a**); washings and salt concentration (**b**); and drying and salt concentration (**c**)

**Fig. 2:**
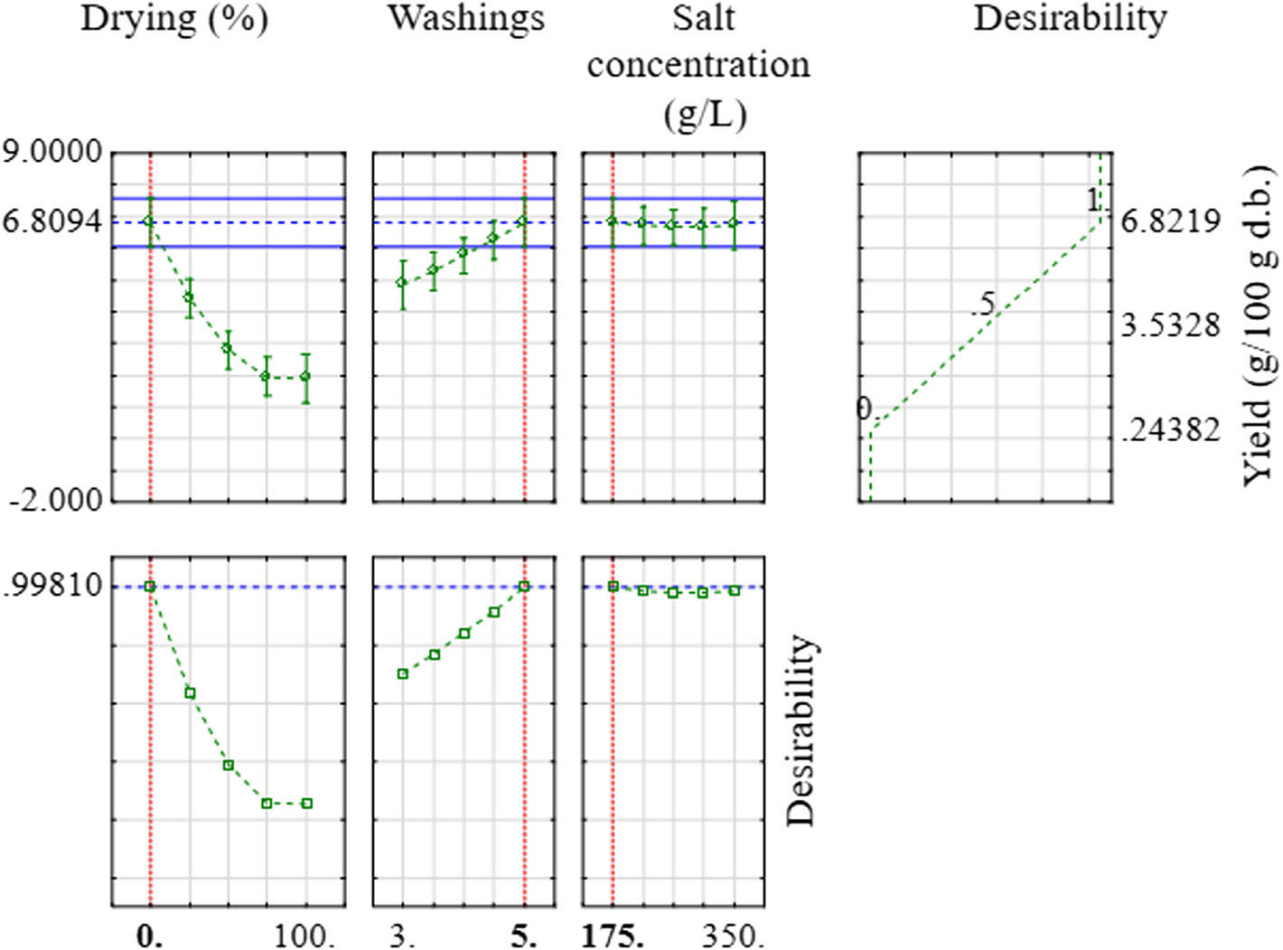
Profiles for predicted values and desirability

The model quality was quantified with the coefficient of determination (R^2^ = 99.44%), the adjusted coefficient of determination (R^2^_A_ = 98.42%), and the root-mean-square error (RMSE = 0.253 g/100 g d.b.). It is clear that the model presents a very interesting quality. Via the analysis of variance test (ANOVA, results not shown), the regression of the corresponding model showed a very highly significant (p <  0.0001), and the lack of fit term showed a not significant difference versus the pure error term (*p* = 0.261 > 0.05). All these results demonstrate that the model is very interesting, very highly significant and valid in the tested experimental domain. Figure 1 presents the fitted essential oil yield as function of different factors. All obtained numerical results were confirmed with Fig. 1. In fact, Fig. 1 shows clearly the importance of drying and washing influences on the yield beside the salt concentration influence. Figure 2 shows also the profiles for predicted values and desirability. The maximum of yield can achieve 6.8 g/100 g d.b. obtained with: without drying, with 5 washings and minimum salt concentration. This was determined with a desirability of 99.81%. This value is very close to those obtained experimentally (condition 3, Table 2).

The essential oil composition was in agree with those reported by Satyal et al. [28] with leaf oil from Nepal, where the amount of monoterpenes exceed the 55% with limonene (20.0%), (E)-phytol (27.5%), linalool (7.0%), 1,8-cineole (6.9%). However, our results disagree with those reported in the literature in both quality and quantity regarding to those obtained by Rahmat et al. [27], where henna leaf oil was dominated by heptadecane constituent (23.5%) followed by tetradecane (16.8%) and hexadecane (14.9%) and phytol (10%). Additionally, Najar et al. [29] had isolated an essential oil from leaves of *Lawsonia inermis* and reported the presence of 80 compounds through GC/MS analysis. The major classes were the apocartenoids (33.6%), followed by the non-terpene derivatives (19.8%), oxygenated sesquiterpenes (12.4%) and monoterpene hydrocarbons (9.9%).

**Fig. 3:**
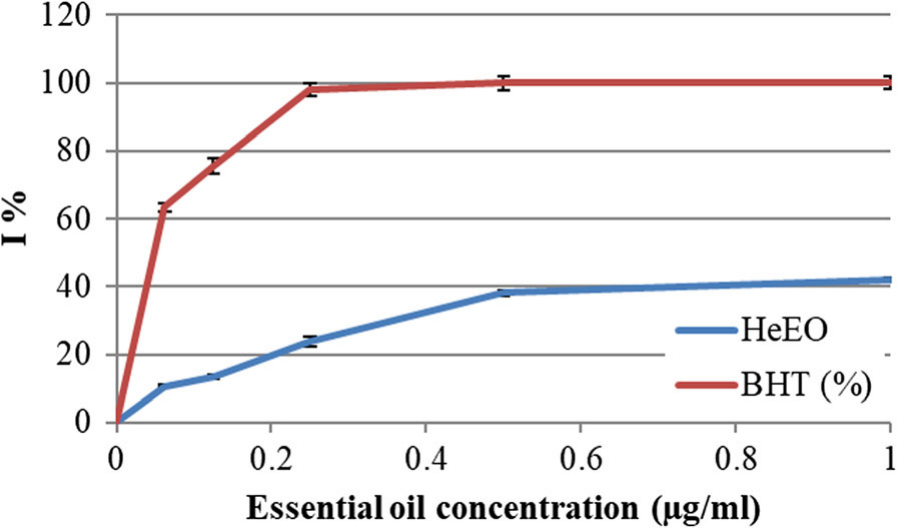
Anti-radical activity against the radical DPPH in inhibition percentage (I %) of the Henna essential oil (HeEO)

The inhibition of percentage of free radical increases with elevated concentration of essential oil and also with BHT (Fig. 3). In fact, the *Lawsonia inermis* essential oil has a substantial anti-radicular effect attending 42%. Therefore, the antioxidant potential of essential oil was so different than phenol extracts, may be due to the difference in chemical structures of phenolic compounds, as suggested by Kedare and Singh [30], as regards the relationship between the chemical structure and antioxidant potential of phenolic compounds by means of the DPPH method.

**Fig. 4:**
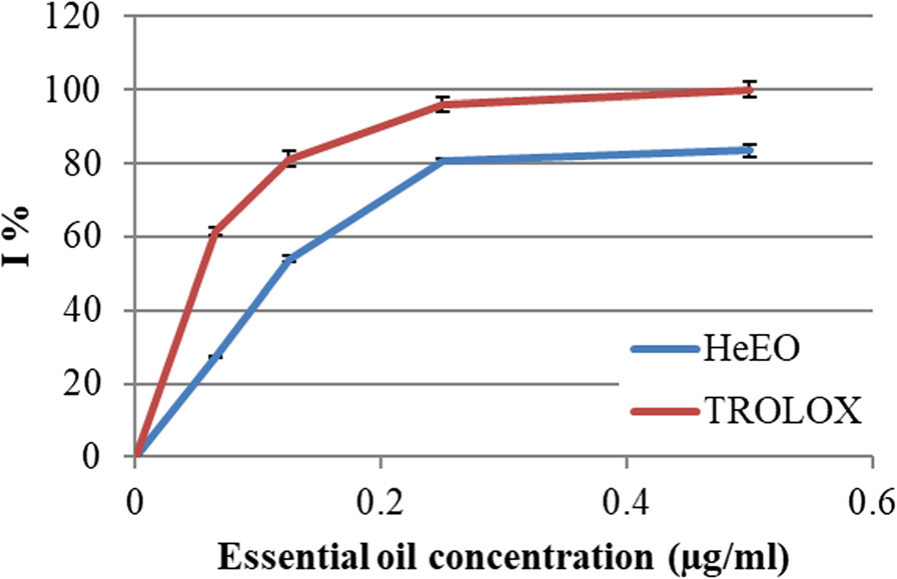
Anti-radical activity against the radical ABTS in percentage of inhibition (I %) of the essential oil of the dried leaves of Henna (HeEO)

Figure 4 shows that the percentage of inhibition of the free radical increases with the increase of concentration, whether for TROLOX or HeEO. Thus, the HeEO seems to be a considerably anti-radical with a percentage inhibition of 80% at a concentration of 500 μg/mL.

The antioxidant activity of henna essential oil can be attributed to hexahydropseudoionone or hexahydrofarnesyl acetone due to their isoprene structures. In fact, Terao [31] found that compounds with basic structure of isoprene, including β-carotene or cartenoids such as canthaxanthin and astaxanthin, exhibited inhibitory effect on the oxidation of methyl linoleate. This might be the reason for essential oil of henna to show better antioxidant activity [31].

At the same meaning; recently work of Khosravi et al. [32] and Carbone et al. [33] show an important antioxidant activity of another Mediterranean essential oil extract from *Origanum vulgare*; which was able to scavenged completely the DPPH radical with only 216 μg/mL of Trolox equivalents. They suggest that this result was related to synergic activity of terpenic and phenolic compounds. This oil shares same compound to henna essential oil, especially as terpenes and phenolic composite.

Moreover, other recent studies, using as Ojewunmi et al. [34], using phenolic compounds extracts from *Lawsonia inermis* have reported a concentration of inhibition at 50% (of DPPH) with 49.22 μg/mL via an ethanolic extract of leaves. Similar work carried by Guha et al. [35] and Enneb et al. [36] reported concentrations of 32.87 μg/mL and 25.73 μg/mL, respectively, of methanolic extracts of this same species. Guha et al. [35] in his phytochemical work on the same extract, reported the order concentrations of 12.59 μg/ mL against the ABTS.

**Fig. 5:**
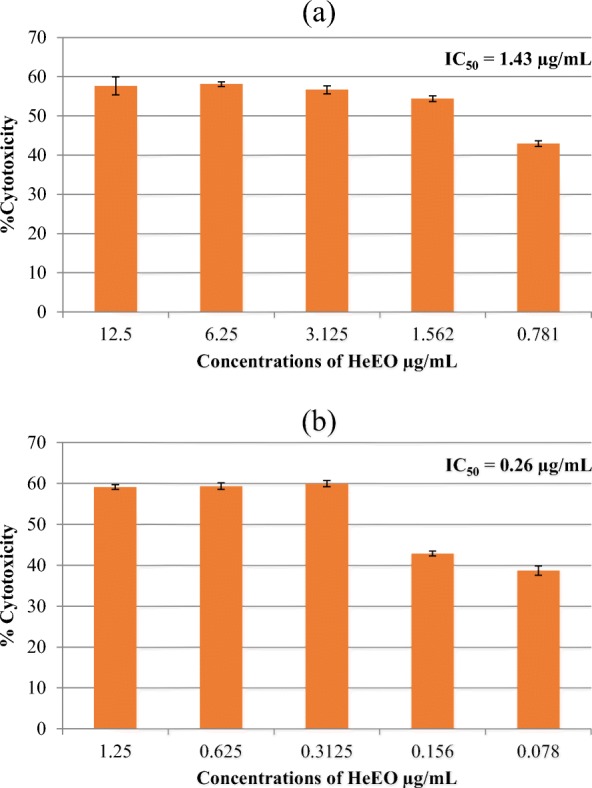
Cytotoxicity of different concentrations of HeEO against HeLa and Raji lines. 240 10^5^ HeLa cells/well (**a**) and Raji cells/well (**b**) are cultured in 96-well plates in the presence of increasing concentrations of oil (Henna). The percentage of cytotoxicity is evaluated by the MTT test

Our results showed a cytotoxic effect dependent concentration of the essential oil of the plant on both cell lines (Fig. 5). The concentration of the tested oil required to reduce the cell survival fraction to 50% of the control was 1.43 μg/mL against HeLa and 0.26 μg/mL against Raji cell line. Rahmat et al. [27] showed an important cytotoxicity effect of leaves essential oil of Henna against HepG2 cell line (IC_50_ = 24 μg/mL). However, the essential oil of *Lawsonia inermis* showed cytotoxic effects on cancer cell lines (MDA-MB-231, MCF7, Chang liver (normal cell) and CaCO_2_). In literature, few articles tested a cytotoxic effect of essential oil of Henna; therefore, many studies explore a diverse solvent extraction and the difference of their effect. Then; Kumar et al. [37] isolated two non-polar fractions from *Lawsonia inermis* with hexane (Hex-LI) and with chloroform (CHCl3-LI) which were explored for their anti-proliferative potential against human cell lines (HeLa, MCF-7, A549 et C6). They showed considerable cytotoxicity effects against HeLa with Hex-LI (IC50 = 382.44 μg/mL) and with CHCL3-LI (IC50 = 741.44 μg/mL). Thus, another research shows that a soluble extract from leaves of *Lawsonia inermis* rich on polyphenolic compounds, induce a moderate anti- proliferative effect on HeLa cell line (minimal dose 31.25 μg/mL inhibit 28.06% of cell line and they noted 61.68% of cytotoxicity with 1000 μg/mL); a comparable effect was also found with MCF-7 cell line [38]. These results corroborate that the essential oil, rich with phenol compounds, proving anti- proliferative potential effect.

To more explain mechanisms of essential oil in general; Bouyahya et al. [39]; show the efficacy of many essential oils according the capacity of to same chemo-types to induce apoptosis when they activate p53 and kinases inhibitor dependent of diverse leukemia and Raji cell line. Then their works demonstrate that these activities were related to terpenique compounds’ as linalool which represents in our case a 7% of “Henna” essential oil.

**Fig. 6:**
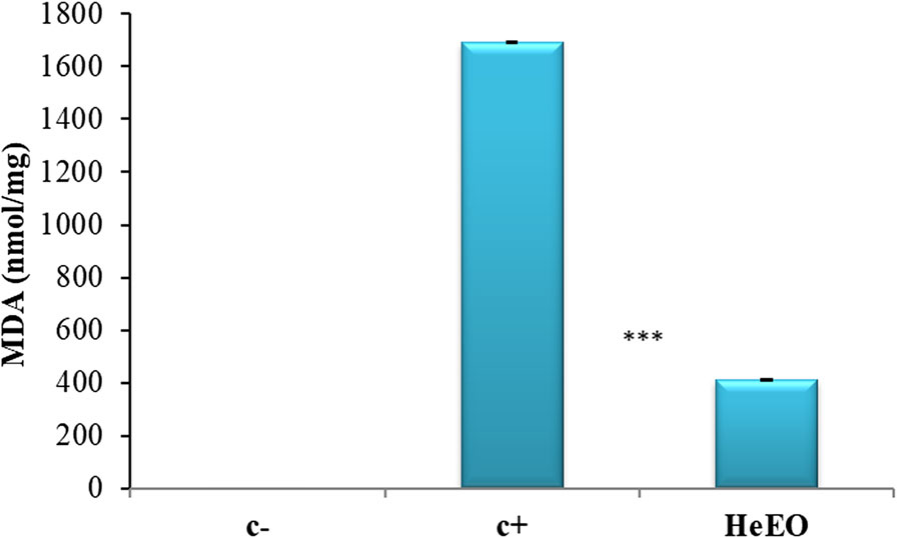
Effect of Henna essential oil on the production of MDA in the Raji line (c-: untreated cells, c+: cells treated with TPA, [HeEO] = 0.01 μg/mL). 3 10^6^ cells are cultured in the presence and absence of TPA and the essential oil extracted from the plant, at a non-cytotoxic concentration. After washing with PBS, the cells are cultured for 48 h. The level of MDA produced is evaluated by the TBARS technique. The results are expressed in nmol/mg of protein (***: *p* <  0.001)

In the cells treated with TPA (12-O-Tetradecanoyl-phorbol-13-acetate), a significant increase in MDA levels was observed at the level of the Raji cell line, compared with the untreated cells (Fig. 6), which indicates the presence of a state of oxidative stress as a consequence of the induction of the EBV lytic cycle (*p* <  0.001). These results are in agreement with the work of Gargouri et al. [24] having shown that the induction of the EBV lytic cycle in the B95–8, Raji and LCL-C1 lines, resulted in an increase in MDA levels and a disruption in the activity of the antioxidant enzymes SOD and CAT. In fact, the concentration of 8 nM of TPA was chosen as the minimum and sufficient concentration to induce the lytic cycle of EBV without inducing a state of oxidative stress [24]. The treatment of Raji cells line with HeEO induces a very significant decrease in MDA levels (*p* <  0.001). This reduced level is probably due to the decrease of lipid peroxidation by the inhibition of the activity of the peroxidase enzyme. The percentage reduction of lipid peroxidation product (MDA) reaches 80% with 0.01 μg/mL HeEO (Fig. 6).

Recently, Kumar et al. [40] have shown following a thiobarbituric lipid peroxidation inhibition test (TBARS) of the butanolic fraction of *Lawsonia inermis* leaves (But-LI), a moderate inhibition of 58.90% at the maximum concentration (1000 μg/mL) and 39.33% at the lowest concentration (100 μg/mL). The But-LI fraction had a higher IC50 (375.73 μg/mL) than the standard Trolox (IC50 = 136.47 μg/mL). Also, some work has published that the commercialized form of *L. inermis* powder, showed not marked inhibition of lipid peroxidation of female albino rats in ascites tumor cells Ehrlich (EAT); that is, the level of MDA in the EAT tested cells did not decrease significantly compared to the untreated control group. However, our results showed a significant difference: HeEO showed a very high potential for free radical scavenging and inhibition of lipid peroxidation, which could be used to counter oxidative stress generated by Raji cells [41].

**Fig. 7:**
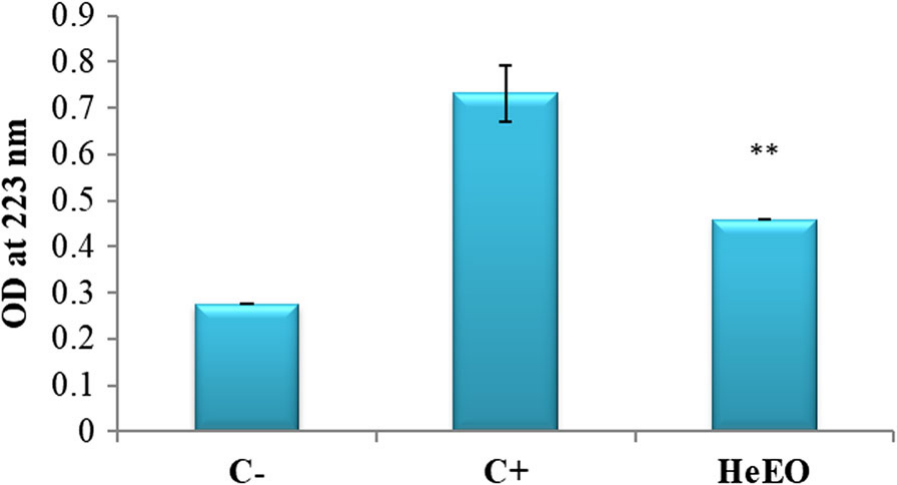
Effect of Henna essential oil on the production of DC in the Raji line (C-: untreated cells, C+: cells treated with TPA, [HeEO] = 0.01 μg/mL). 3 10^6^ cells are cultured in the presence and absence of TPA and the essential oil extracted from the plant, at a non-cytotoxic concentration. After washing with PBS, the cells are cultured for 48 h. The level of DC produced is evaluated by measuring the OD at 233 nm (*: *p* < 0.05)

Treatment of the Raji cell line with TPA induced a significant increase in CD levels compared to untreated cells (*p* <  0.005) (Fig. 7). Our results are in agreement with those of Gargouri et al. [42] having shown that the induction of the lytic cycle of EBV in the B95–8 and Raji lineages led to an increase in CD levels [42] Notably, Raji cells treated with Henna essential oil exhibited a very significant antioxidant effect compared to the control with 40% total inhibition. Decreases level of CD were observed with the used concentration (0.01 μg/mL), compared with untreated cells (p <  0.005). This, which proves the capacity of this essential oil to reduce the lipid peroxydase activity and then a quantity of CD.

The result of this study assumed a potential relationship between the cytotoxic effect against Raji cell line and antioxidant activity, and also anti-peroxides’ activity. Antioxidants are known to relieve oxidative stress, which is generally supposed as one of mutations in genome; then antioxidants are thought to provide protection against cancer [43].

## Conclusion

The essential oil of *Lawsonia inermis* L. showed promising effects on inhibition cancer cell lines and its use could be commercialized as chemotherapeutic supplement due to its significant antioxidant activity, lipid peroxidation inhibition activity and restraining cell line cancer proliferation. Extensive investigation is needed to exploit their therapeutic utility to battle same diseases’ drug with compound isolated from henna can be explored more their potential bioactivity and to elucidate their mechanism action. However in vivo experiences let be necessary to more validate this deduction.

## Data Availability

The dataset supporting the conclusions of this article is included within the article.

## Abbreviations

*p*: Value of probability (dimensionless)
R^2^: Determination coefficient (dimensionless)
R^2^_A_: Adjusted determination coefficient (dimensionless)
RMSE: Root-Mean-Square Error
t: Student coefficient (dimensionless)
Y: extraction yield (%)
